# Cold-Rolled Strip Steel Stress Detection Technology Based on a Magnetoresistance Sensor and the Magnetoelastic Effect

**DOI:** 10.3390/s18051638

**Published:** 2018-05-21

**Authors:** Ben Guan, Yong Zang, Xiaohui Han, Kailun Zheng

**Affiliations:** 1 School of Mechanical Engineering, University of Science and Technology Beijing, Beijing 100083, China; guanben@ustb.edu.cn or niuben57@163.com (B.G.); xiaohuihan1@163.com (X.H.); 2 Department of Mechanical Engineering, Imperial College London, Exhibition Road, London SW7 2AZ, UK; k.zheng13@imperial.ac.uk

**Keywords:** magnetoelastic effect, magnetoresistance sensor, cold rolled strip, stress detection

## Abstract

Driven by the demands for contactless stress detection, technologies are being used for shape control when producing cold-rolled strips. This paper presents a novel contactless stress detection technology based on a magnetoresistance sensor and the magnetoelastic effect, enabling the detection of internal stress in manufactured cold-rolled strips. An experimental device was designed and produced. Characteristics of this detection technology were investigated through experiments assisted by theoretical analysis. Theoretically, a linear correlation exists between the internal stress of strip steel and the voltage output of a magneto-resistive sensor. Therefore, for this stress detection system, the sensitivity of the stress detection was adjusted by adjusting the supply voltage of the magnetoresistance sensor, detection distance, and other relevant parameters. The stress detection experimental results showed that this detection system has good repeatability and linearity. The detection error was controlled within 1.5%. Moreover, the intrinsic factors of the detected strip steel, including thickness, carbon percentage, and crystal orientation, also affected the sensitivity of the detection system. The detection technology proposed in this research enables online contactless detection and meets the requirements for cold-rolled steel strips.

## 1. Introduction

Cold-rolled strip steel, as an important industrial raw material, has been extensively applied in the automotive, home appliance, hardware, and other important manufacturing industries. The manufacture of cold-rolled strip steel requires strong and advanced technologies in the steel industry to produce products with high quality and geometric accuracy [1]. The shape of the strip is an important influencing factor determining the quality of cold-rolled strip steel. Basically, the shape quality of the strip refers to the degree of wave, buckling, or other flatness defects in strip steel products. From the material mechanics viewpoint, these defects result from non-equal ductility along the width direction of the strip steel during the production process. The unbalanced internal stress arising from the non-equal ductility of the material in the width direction resulted in defects in the strip shape [2,3,4]. To precisely control shape defects, a certain amount of tensile stress is normally exerted along the longitudinal direction of strip steel during production. Subsequently, through the detection of variations in internal stress at different locations in the width direction, the ductility differences can be obtained correspondingly to help the control the final strip shape using relevant methods [5,6]. Hence, an accurate measurement of the internal stress under the strip steel tension effect becomes the most important detection stage to control strip shape.

The internal stress detection device extensively used for industrial production of strip steel is a kind of contact detection roller [7,8]. With this methodology, a significant amount of pressure sensors are mounted on the surface of the detection roller, which capture pressures exerted on this detection roller by the tension at all positions of the strip steel. Then the internal stress of the strip steel can be calculated using pressure data. Such a contact detection device has a high detection precision advantage. However, the surface of strip steel is easily scratched by the device during detection. In addition, the roller surface of this device must be reground once wear occurs, resulting in the pressure sensor needing to be re-calibrated. The high cost and maintenance requirements are driving engineering areas to develop a low-cost and highly accurate contactless detection technology, believed to be the core technology focus for the next generation internal stress detection devices for strip steel.

Many investigations into fundamental theories and applications have been performed on contactless detection of internal stresses of ferromagnetic materials using the magnetic survey method. From the aspect of theoretical contributions, Yamada et al. [9] and Wakiwaka et al. [10,11] comprehensively analyzed the working principle of a quadripolar sensor based on the magnetoelastic effect. The research concluded that magnetic permeability distributed in an elliptical profile in all directions of principal stresses linearly varies with principal stress. Liu et al. [12] thoroughly used circuit theory, magnetization theory, stress analysis theory, as well as the law of electromagnetic induction and obtained quantitative equations of the output voltage and principal stress difference for quadripolar sensors based on the magnetoelastic effect. Kypris et al. [13] established a correlation function between the frequency spectrum of Barkhausen noise and the bending moment of ferromagnetic material, such a relation function was able to be used to characterize the residual stress-depth profiles of ferromagnetic specimens. In terms of applications, Jackiewicz [14] evaluated the stress state for steel truss structures using the magneto-elastic effect detection method. Vourna et al. [15] detected the residual stress of welding structures based on the Barkhausen effect. In addition, Zhang [16] and Yu et al. [17] conducted theoretical and experimental research on internal stress detection method for cold-rolled strips based on the magnetoelastic effect, which was motivating research for this study.

At present, the magnetic detection stress method is based on alternating magnetic fields. In this method, a magnetic core is used as the excitation coil and the other magnetic core serves as a detection coil. Alternating Current (AC) with certain magnitude and frequency can be charged into the excitation coil to excite ferromagnetic materials to generate an alternating magnetic field. The magnetoconductivity of the ferromagnetic material varies due to the influence of internal stress, which subsequently induces variations in magnetic flux density. The signal of these magnetic flux variations can be detected by the induction coil and used to obtain the internal stress of materials. Although the detection method can detect internal stress for ferromagnetic materials without contact, inspection depth of internal stress is limited due to the approaching skin effect initiated by using alternating magnetic fields. Because AC signals have weak anti-interference and signal processing capacities, and sensitivity and resolution of the induction coil are low, complicating the accurate measurement of internal stress with slight changes, in which the magnetic detection stress method is mainly used currently for detecting welding residual stress with large variations in internal stress.

To address the problems mentioned above, this study proposes a stress detection technology based on a magneto-resistance sensor and the magnetoelastic effect for cold-rolled strip steel. An experimental device based on this detection mechanism was designed and established to characterize the detection capabilities of this technology. The characteristics of the detection technology were discussed thoroughly using theoretical analysis and experimentation. The results showed that using a steady-state magnetic field as the excitation field of ferromagnetic materials contributes to obtaining a greater detection depth of internal stress and facilitates signal processing. In addition, using a magnetoresistive sensor with high sensitivity and resolution to capture weak variations in magnetic induction contributes to improving sensitivity and resolution of internal stress detection, which can satisfy industrial requirements for accurate detection of internal stress for strip steel.

## 2. Stress Detection Principle and Theoretical Analysis

### 2.1. Basic Detection Principle

The magnetoelastic effect mainly refers to the phenomenon whereby the magnetic permeability and magnetic reluctance of ferromagnetic materials vary with loading of external forces, such as tension, compression, and twisting force, which results in the generation of magnetic anisotropy. This characteristic causes the magnetic permeability in ferromagnetic materials in a constant weak magnetic field to change under external stresses, resulting in a slight change in magnetic flux density around ferromagnetic materials. Correspondingly, a correlation between variations in internal stress state and slight changes of the surrounding magnetic flux density can be established [18]. The magneto-resistive effect refers to the change in material resistance due to the magnetic field, which can be mainly divided into anisotropic magnetoresistance (AMR), giant magnetoresistance (GMR), and tunneling magnetoresistance (TMR) according to different magnetoresistance effect principles. Detection capability of sensors using the magneto-resistor has significantly improved since the giant magneto-resistive effect was first discovered in (Fe/Cr)N multilayer film material in 1988 [19]. Many detection technologies based on the magneto-resistive sensor have been applied successfully [20,21,22,23,24], making possible the direct detection of weak changes in magnetic induction caused by magnetoelastic effect.

**Figure 1:**
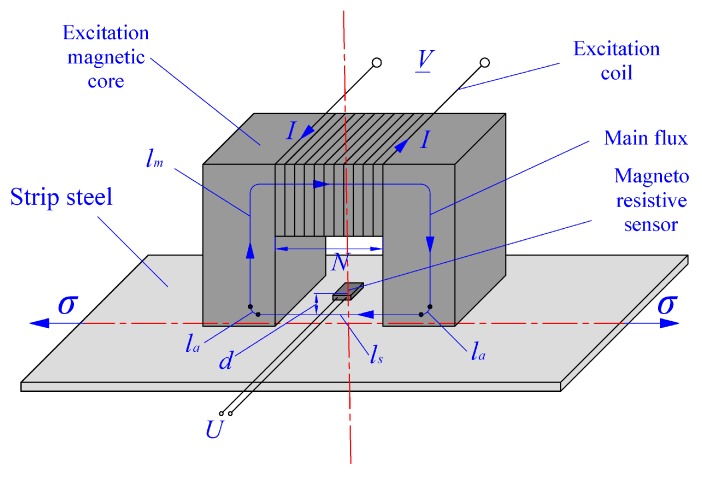
Basic schematic diagram for stress detection of strip steel.

Based on above analysis, the basic principle for stress detection of strip steel proposed in this paper involves: first, an excitation coil with a charged direct current (DC) is used to provide a steady-state weak magnetic field for the detected strip steel. Then, changes in internal stress within the strip steel exposed to the steady-state weak magnetic field can lead to a change in its magnetic permeability. Subsequently, the internal magnetic flux density inside the strip steel varies and the magnetic flux density around this strip steel exhibits slight variations. Finally, these slight changes are directly converted to electrical signals using a highly sensitive magneto-resistive sensor, and the internal stress state of strip steel is obtained. The sequence of signal conversion can be expressed as: Δ*σ→*Δ*μ→*Δ*Bs→*Δ*Br→*Δ*R→*Δ*U*, where Δ*σ* represents the variation in the internal stress of strip steel, Δ*μ* represents the variation in the magnetic permeability of strip steel under the effect of stress, Δ*Bs* represents the variation in the magnetic flux density for strip steel caused by changes in magnetic permeability, Δ*Br* is the variation in magnetic flux density of the sensor inspection position caused by variation in the magnetic flux density of strip steel, Δ*R* represents the variation in sensor resistance value caused by the change in magnetic flux density, and Δ*U* is the variation in voltage finally output by the sensor. Figure 1 shows the basic detection principle. The symbols used in this figure will be explained in Section 2.2.

According to the analysis of signals conversion, a magneto-resistive sensor can directly capture variations in magnetic induction around the strip steel, and then convert them into electrical signals. Therefore, an alternating magnetic field to induce the detection coil to generate electromotive force and output signal is not required. In this situation, variations in the internal stress for strip steel can be detected through a steady-state magnetic field, effectively avoiding the “skin effect” of alternating magnetic fields. The detection depth for internal stress is increased, and the anti-jamming signal processing capacities are significantly improved. Conversely, compared to induction coils, detection precision and sensitivity of magneto-resistive sensors for weak magnetic fields can be greatly improved, so the sensitivity and resolution of detecting internal stress are improved accordingly.

### 2.2. Relationships in Signal Conversion

To obtain the relationship between the detected Δ*σ* and output signal Δ*U*, the relationship between two individual signals during each signal conversion must be established according to the sequence of signal conversion Δ*σ→*Δ*μ→*Δ*Bs→*Δ*Br→*Δ*R→*Δ*U* as discussed earlier. In addition, corresponding influencing parameters must be identified and analyzed.

#### 2.2.1. Δ*σ→*Δ*μ*

Wang et al. [25] indicated that Δ*σ* and Δ*μ* are in a near-linear relationship when elastic deformation is experienced by ferromagnetic materials, and weak magnetic fields are much lower than the saturated magnetic flux density. The relationship can be expressed as follows:

(1) $$\Delta \mu =k_{\sigma -\mu }\Delta \sigma$$

where $k_{\sigma -\mu }$ indicates the inherent attribute of ferromagnetic material for strip steel, which is related to the saturated magnetic flux density $B_{m}^{\prime }$, the saturated magneto-strictive coefficient $\lambda_{m}$, and magnetic permeability $\mu_{0}$ under unstressed conditions of strip steel materials. Equation (1) is the common theoretical principle for all internal stress detection technologies using the magneto-elastic effect.

#### 2.2.2. Δ*μ→*Δ*Bs*

The relationship between Δ*μ* and Δ*Bs* can be obtained according to the definition of magnetic flux density, as shown in Equation (2):

(2) $$\Delta B_{s}=H_{s}\Delta \mu$$

Accordingly, the conversion coefficient between Δ*Bs* and Δ*μ* is the magnetic field intensity of strip steel, $H_{s}$. The specific expression of $H_{s}$ is very complex. Its influencing factors can be analyzed using the Ampere’s circuital theorem. Assuming all magnetic induction lines with the magnetic field extend along the main flux direction, a closed complete curve penetrating the magnetic gap and strip steel along the center line of the magnetic core can be drawn, which is regarded as the integration path of the Ampere ring road (Figure 1). The number of excitation coils is set as *N*; the exciting current is I. $H_{s}$, $H_{a}$, and $H_{m}$ indicate the magnetic field intensity values caused by the main flux in strip steel, air, and magnetic core, respectively. $l_{s}$, $l_{a}$, and $l_{m}$ indicate the effective length of a magnetic circuit in strip steel, air, and magnetic core, respectively. According to Ampere circuital theorem:

(3) $$\oint_{L_{1}}\vec{H}\cdot d\vec{r}=\int_{l_{s}}H_{s}dr+\int_{l_{a}}H_{a}dr+\int_{l_{m}}H_{m}dr=NI$$

which can be rewritten as:

(4) $$NI=H_{s}l_{s}+H_{a}l_{a}+H_{m}l_{m}$$

The magnetic induction and magnetic permeability in the air and magnetic core are represented by $B_{a}$, $B_{m}$, $\mu_{a}$ and $\mu_{m}$ respectively. Then, according to Equation (2), substituting $H_{a}=B_{a}/\mu_{a}$ and $H_{m}=B_{m}/\mu_{m}$ into Equation (4). The $H_{s}$ can be expressed as:

(5) $$H_{s}=\frac{NI-B_{a}l_{a}/\mu_{a}+B_{m}l_{m}/\mu_{m}}{l_{s}}$$

Equation (5) shows that the conversion coefficient $H_{s}$ is mainly related to the number of excitation coils *N*, current *I*, material and structure parameters $B_{m}$, $\mu_{m}$, and $l_{m}$ of the magnetic core, and the clearance $l_{a}$ between the magnetic core and strip steel. However, Equation (5) is not an accurate expression of the magnetic field intensity of strip steel.

#### 2.2.3. Δ*Bs→*Δ*Br*

**Figure 2:**
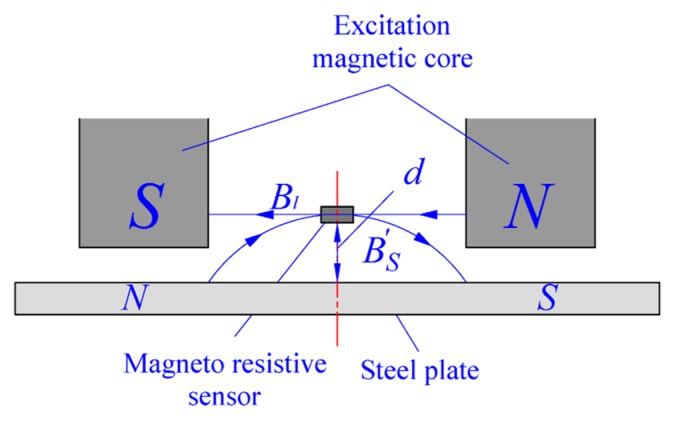
Schematic diagram of magnetic induction for sensor detection position.

As can be seen in Figure 2, the magnetic induction *Br* of the sensor detection position can be treated as a vector sum of the magnetic induction $B_{l}$, formed by the leaked flux of the excitation field and magnetic induction $B_{S}^{\prime }$ generated around the magnetized strip steel. $B_{l}$ and $B_{S}^{\prime }$ have completely opposite direction according to the direction of the magnetic induction line when the sensor is placed on the center line of magnetic core. Then:

(6) $$B_{r}=B_{l}-B_{S}^{\prime }$$

The magnetic induction $B_{l}$ formed by the leakage flux of the excitation field is basically a constant according to the electro-magnetism theory. The relationship between the magnetic induction of strip steel $B_{S}$ and $B_{S}^{\prime }$ can be approximately assumed to be that between the surface magnetic induction of a rectangular electric coil with finite length and its environmental magnetic induction. According to the literature [26,27,28], within a certain distance approaching the surface of an electric coil, the magnetic induction decreases exponentially with approximately increasing the distance, which is expressed as:

(7) $$B_{S}^{\prime }=(a_{s-s^{\prime }}{)}^{d}B_{s}$$

where $a_{s-s^{\prime }}$ is a coefficient related to the distribution of the magnetic field generated by the strip steel. Since the magnetic field of strip steel results from the excitation magnetic field, its influencing factors are consistent with those of an excitation magnetic field. *d* is the vertical distance between the detection point and strip steel, as shown in Figure 2. Therefore, *B_r_* can be expressed as $B_{r}=B_{l}-{\left(a_{s-s^{\prime }}\right)}^{d}B_{s}$. The increase in $B_{r}$ after differentiation is:

(8) $$\Delta B_{r}=-{\left(a_{s-s^{\prime }}\right)}^{d}\Delta B_{s}$$

Therefore, the conversion coefficient between $\Delta B_{r}$ and $\Delta B_{s}$ is an exponential function of vertical distance d between the detection position of sensor and strip steel plane.

### 2.2.4. *ΔBr→ΔR→ΔU*

**Figure 3:**
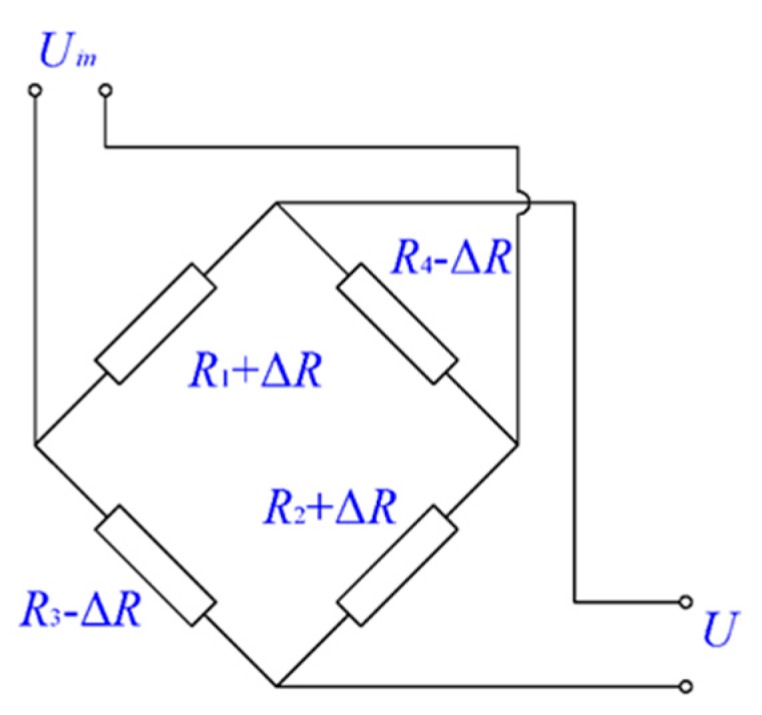
Schematic circuit diagram for a magneto-resistive sensor.

The signal conversion process of *ΔBr→ΔR→ΔU* is completed within the magneto-resistive sensor. The general internal structure of GMR and TMR sensors is Wheatstone bridge (Figure 3), which includes four magnetoresistance sensors with a same resistance. The relationship between the resistance value of the magnetoresistance sensor *ΔR* and Δ*Br* is given by Equation (9):

(9) $$\Delta R=k_{B_{r}-R}\Delta B_{r}$$

where $k_{B_{r}-R}$ indicates the conversion coefficient between Δ*Br* and Δ*R*, which is determined by the type and performance of the magnetoresistance sensor used in the sensor.

Figure 3 shows a circuit diagram for a magneto-resistive sensor, showing that the values of the four resistances are the same, *R_1_* = *R_2_* = *R_3_* = *R_4_* = *R*, for a zero magnetic field. If a magnetic field is applied, resistances *R_1_* and *R_2_* can be increased to *R_1_* = *R_2_* = *R* + Δ*R*, whereas resistances *R_3_* and *R_4_* can be decreased to *R_3_* = *R_4_* = *R* − Δ*R*. The magnetic field is obtained according to the Wheatstone bridge property that states the relationship between output signal Δ*U* and Δ*R* as:

(10) $$\Delta U=\frac{U_{in}}{R}\Delta R$$

where $U_{in}$ indicates the supply voltage of Wheatstone bridge and R indicates the resistance value of the magnetoresistance sensors. Thus, the conversion coefficient between Δ*R* and Δ*U* is $U_{in}/R$.

The conversion coefficient and influential parameters in all previous signal conversion processes can be obtained using the abovementioned analysis, which is summarized in Table 1.

### 2.3. Analysis of Stress Detection Sensitivity

The relationship between detected Δ*σ* and output signal Δ*U* can be established according to Section 2.2, allowing the sensitivity and influence factors of stress detection to be discussed. Combining Equations (1), (2) and (8)–(10), the relationship between Δ*σ* and Δ*U* can be obtained as:

(11) $$\Delta U=-\frac{H_{s}{\left(a_{s-s^{\prime }}\right)}^{d}U_{in}k_{B_{r}-R}}{R}k_{\sigma -\mu }\Delta \sigma$$

Therefore, sensitivity *k* of strip steel in the stress detection process is:

(12) $$k=\frac{\Delta U}{\Delta \sigma }=-\frac{H_{s}{\left(a_{s-s^{\prime }}\right)}^{d}U_{in}k_{B_{r}-R}}{R}k_{\sigma -\mu }$$

Besides the parameter $k_{\sigma -\mu }$, which is determined by intrinsic magneto-elastic characteristics of strip steel, the other parameters can be actively designed and adjusted based on the stress detection requirements. For example, to increase the sensitivity of the stress detection system, approaches such as increasing exciting current *I* to increase magnetic field intensity $H_{s}$, properly increasing supply voltage $U_{in}$ of the sensor, or decreasing sensor detection point and shortening the vertical distance of strip steel *d*, can be used.

### 2.4. Relationship of Relative Orientations between Stress Directions, Main Flux Direction and Sensor Detection Directions

The sensitivity of the detection system is also affected by relative orientation relationships among the stress direction of the strip steel, the main flux direction of the excitation field, and the detection direction of the magneto-resistive sensor in the detection process, which must be analyzed thoroughly to identify the optimal relative positions to obtain the highest detection sensitivity.

**Figure 4:**
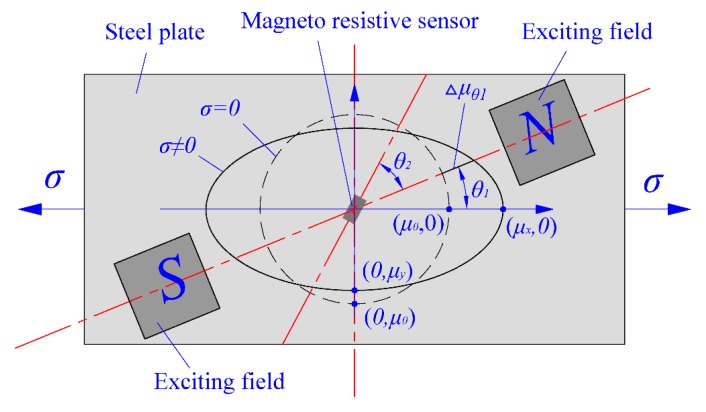
Influences of the relative orientation relationship of stress direction, main flux direction, and detection direction of a sensor on sensitivity.

According to real-life industrial strip steel production, the internal stress state during detection process is always maintained in a uniaxial tensile state in the longitudinal direction of the strip steel. So, taking the longitudinal direction of strip steel as the base orientation, assume the included angle between the main flux direction of the excitation field and strip steel length is $\theta_{1}$, and the included angle between the detection direction of the magneto-resistive sensor and the main flux direction of the excitation field is $\theta_{2}$. According to the findings in the literature [9,10], the magnetic permeability of ferromagnetic materials is uniformly distributed in a circular profile in all directions under an unstressed condition. Its expression is described in Equation (13), where $\mu_{0}$ indicates the magnetic permeability under an unstressed condition. Magnetic permeability of ferromagnetic materials is distributed in an ellipse profile under uniaxial tensile stress conditions. Its expression is described in Equation (14), where $\mu_{x}$ and $\mu_{y}$ indicate magnetic permeability in the long axis and short axis direction of ellipse, respectively (Figure 4):

(13) $$\{\begin{array}{c} x=\mu_{0}\cos\theta \\ y=\mu_{0}\sin\theta \end{array}\;\left(0<\theta <2\pi \right),$$

(14) $$\{\begin{array}{c} x=\mu_{x}\cos\theta \\ y=\mu_{y}\sin\theta \end{array}\;\left(0<\theta <2\pi \right),$$

Variations in magnetic permeability $\Delta \mu_{\theta_{1}}$, in direction $\theta_{1}$ is the linear distance between point $\left(\mu_{x}\cos\theta_{1},\mu_{y}\sin\theta_{1}\right)$ and point $\left(\mu_{0}\cos\theta_{1},\mu_{0}\sin\theta_{1}\right)$, which is:

(15) $$\Delta \mu_{\theta_{1}}=\sqrt{\mu_{x}{}^{2}\cos^{2}\theta_{1}+\mu_{y}{}^{2}\sin^{2}\theta_{1}}-\mu_{0}$$

Combining Equations (2), (8) and (15), the variation in magnetic induction $\Delta B_{r\theta_{1}}$, in direction $\theta_{1}$ at the detection position of sensor can be obtained, which is:

(16) $$\Delta B_{r\theta_{1}}=-{\left(a_{s-s^{\prime }}\right)}^{d}H_{s}(\sqrt{\mu_{x}{}^{2}\cos^{2}\theta_{1}+\mu_{y}{}^{2}\sin^{2}\theta_{1}}-\mu_{0})$$

As a magneto-resistive sensor can only be used to measure magnetic flux density in the detection direction, the actual detected variation in magnetic flux density $\Delta B_{r}（\theta_{1},\theta_{2})$ is given in Equation (17), when the included angle between the detection direction of the magneto-resistive sensor and $\Delta B_{r\theta_{1}}$ is $\theta_{2}$.

(17) $$\Delta B_{r}（\theta_{1},\theta_{2})=\Delta B_{r\theta_{1}}\cos\theta_{2}=-{\left(a_{s-s^{\prime }}\right)}^{d}H_{s}(\sqrt{\mu_{x}{}^{2}\cos^{2}\theta_{1}+\mu_{y}{}^{2}\sin^{2}\theta_{1}}-\mu_{z})\cos\theta_{2}$$

As can be seen in Equation (17), the extreme points of $\Delta B_{r}（\theta_{1},\theta_{2})$ are (0,0) and (π/2,0) with correspondingly extreme values of $-{\left(a_{s-s^{\prime }}\right)}^{d}H_{s}(\mu_{x}-\mu_{0})$ and $-{\left(a_{s-s^{\prime }}\right)}^{d}H_{s}(\mu_{y}-\mu_{0})$. $\left(\mu_{y}-\mu_{0}\right)$ is generated by the influence of transverse strain of strip steel materials, which is not stable in the detection process. In addition, $\left(\mu_{y}-\mu_{0}\right)$ is much smaller than $\left(\mu_{x}-\mu_{0}\right)$, thus the largest extreme point of $\Delta B_{r}（\theta_{1},\theta_{2})$ is (0,0), which indicates that the maximum sensitivity of stress detection is obtained when the main flux direction of the exciting field, the detection direction of magneto-resistive sensor, and the length direction of strip steel are consistent during the detection process.

## 3. Establishment of Stress Detection System

### 3.1. Experimental Set-Up

**Figure 5:**
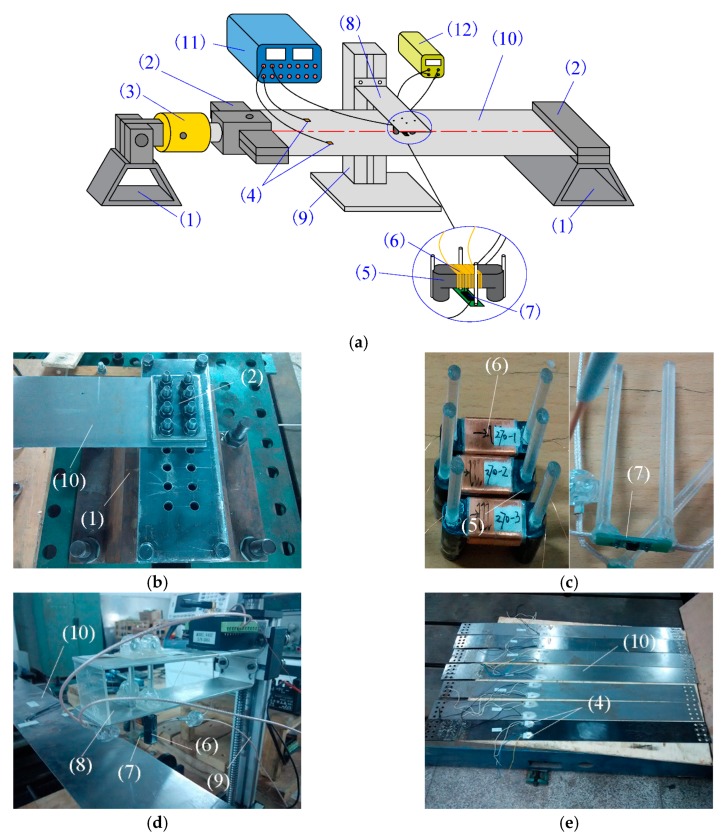
Composition and structure of experimental device for stress detection: (**a**) Structure diagram of experimental device; (**b**) Fixed method of detected strip steel; (**c**) Exciting magnetic core and magneto-resistive sensor; (**d**) Installation method of exciting magnetic core and magneto-resistive sensor; (**e**) Detected strip steel and strain gage. The device components include: (1) supporting frames, (2) clamping plates, (3) hydraulic cylinder, (4) strain gauges, (5) magnetic core, (6) excitation coil, (7) magneto-resistive sensor, (8) supporting plate, (9) guide rail, (10) strip steel, (11) data acquisition instrument, and (12) power supply.

To verify the feasibility of the proposed stress detection principle and explore the effects of external parameters, an experimental device for stress detection based on a magneto-resistive sensor and converse magnetostrictive effect was designed and established, as shown in Figure 5a. The device is composed of a stress loading system, stress detection system, detected strip steel, data acquisition instrument, and power supply.

The stress loading system is composed by two supporting frames, two clamping plates, a double-action hydraulic cylinder, and two strain gauges. The detected strip steel was fixed using two clamping plates on the supporting frame and hydraulic cylinder by bolting as shown in Figure 5b. The required internal tensile stress was generated within the detected strip steel by controlling the stroke of the piston rod of the hydraulic cylinder. The magnitudes of the induced internal stresses were calculated using the two strain gauges mounted on the symmetrical positions on the two sides of the center line of the detected strip steel. The strain data were recorded using the data acquisition instrument, which was used to determine internal stress. The consistency of strain values was used to characterize whether the strip steel was uniformly stressed, which was adjusted by the fastening force of the bolts on the clamping plates so the strip steel could sustain uniform tensile stress along the width direction.

The stress inspection system included a, exciting magnetic core, excitation coil, magneto-resistive sensor, supporting plate, and a guide rail. The excitation coil was twined around the exciting magnetic core. The exciting magnetic core and magneto-resistive sensor were fixed on the supporting frame using a plastic rod and glass cement. Relative positions of the exciting magnetic core, magneto-resistive sensor and the distances with the detected strip steel were unchanged, as shown in Figure 5c,d. The support was fixed on the guide rail and was able to be moved vertically along the guide rail to adjust distances between the exciting magnetic core, magneto-resistive sensor, and detected strip steel. The power supply was used to provide constant current for the excitation coil and constant voltage for the magneto-resistive sensor. The stress detection signal of the magneto-resistive sensor was input into the data acquisition instrument for recording and display.

### 3.2. Design of Exciting Parameter and Model Selection of Magneto-Resistive Sensor

The steady-state magnetic field formed by exciting parameter must be a weak magnetic field for detected strip steel using experimental device, which makes the strip steel below the saturation induction, and maintains an approximately linear Δ*σ* and Δ*μ* relationship, to ensure the approximately linear relation between input and output signals. The sensitivity and resolution of selected magneto-resistive sensors must guarantee that weak magnetic induction within the stress detection range can be identified and captured, which contributes to ensuring that the whole system has sufficient sensitivity and resolution for internal stress detection of strip steel.

**Figure 6:**
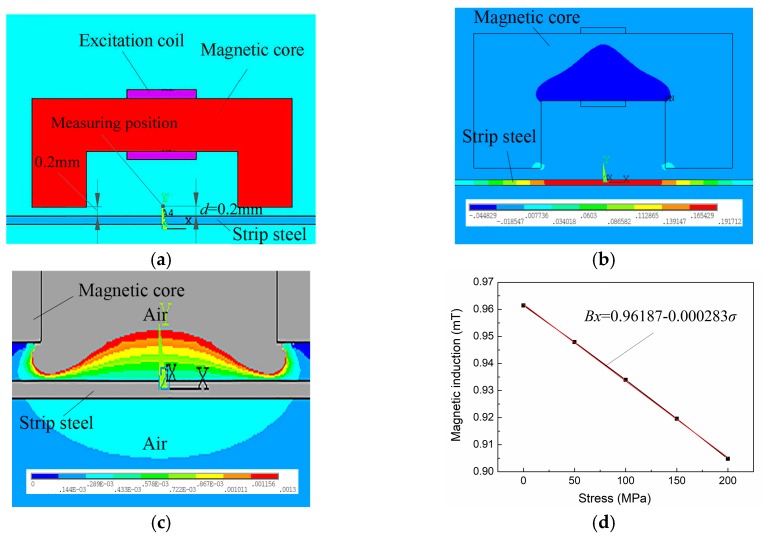
Numerical simulation analysis of exciting parameters: (**a**) Finite element model; (**b**) distribution of magnetic induction within detected strip steel; (**c**) magnetic induction surrounding detected strip steel; and (**d**) change in magnetic induction for detected point with stress.

U22 manganese zinc ferrite was used as the exciting magnetic core for testing. The number of turns of the excitation coil *N* was 450. Enameled copper wire with a diameter of 0.02 mm was used. A 200 mA steady current was provided. To determine exciting parameters and verify whether they can satisfy detection requirements, ANSYS finite element analysis software was used preliminarily to evaluate strip steel, surrounding magnetic induction, and variation range of magnetic induction for magneto-resistive sensor detection position under stress effect within group work. A two-dimensional (2D) mechanical magnetic coupling model was established, as shown in Figure 6a. 

The material used as the detection candidate was Q235 (E235B in ISO630). The thickness was 1.0 mm. The mechanical and magnetic characteristic parameters are provided in Table 2. 

Relative permeability of air and excitation coil in the setting model was 1; magnetic permeability of excitation coil was 2000. To conduct a pure electromagnetic field simulation, mechanical-magnetic coupling was subject to indirect coupling [29], which converts numerical change in internal stress of strip steel into the numerical change in magnetic permeability $\mu_{\sigma }$ for materials with Equation (18) [25]:

(18) $$\mu_{\sigma }=\frac{B_{m}^{2}+\sqrt{B_{m}^{4}-8\sigma \lambda_{m}\mu_{0}B_{m}^{2}}}{4\sigma \lambda_{m}\mu_{0}}$$

The distribution of the internal magnetic induction for detected strip steel can be obtained through numerical simulation, as shown in Figure 6b, which indicates that internal magnetic induction for detected strip steel gradually decreases from the center line of the exciting magnetic core to magnetic pole. The maximum magnetic induction of the exciting parameter generated in the strip steel was 0.192 T, which was much less than the 2.5 T saturation induction for steel plate and below the near-linear section of Δ*σ* and Δ*μ* relationship of Q235 steel. Thus, the exciting parameters ensures detected strip steel satisfies the weak magnetic field condition.

The distribution of the magnetic induction surrounding strip steel is shown in Figure 6c, which shows that the magnetic induction surrounding strip steel ranges from 0.2 to 1.3 mT and increases with the increase in the vertical distance *d* of strip steel. The distance *d* between the detection point of the sensor and the strip steel was set to 2.0 mm, variations in the magnetic induction for the detection point with stress variations is shown in Figure 6d, which indicates that the magnetic induction for the detection point decreases linearly with the increase in internal stress. Through the linear fitting of the simulated data, the conversion coefficient between the variable quantity Δσ of internal stress and the variable quantity $\Delta B_{s}$ of the magnetic induction for the detection point was 0.000283 mT/MPa (2.83 mOe/MPa).

Based on above-mentioned analysis, the TMR2102 magneto-resistive sensor was preliminarily selected as a sensor for detection. Its performance parameters are shown in Table 3. The variation range in magnetic induction is completely covered by its linear region ±3.0 mT. The input voltage of the sensor in experimental process was 5 V and the sensitivity of the sensor was 245 mv/mT (49 mv/v/mT × 5 V), thus the detection sensitivity of the sensor to stress was about 0.07 mv/MPa (0.00283 mT/MPa × 245 mv/mT). 

To ensure the data acquisition instrument could conduct accurate A/D conversion for output signals of the sensor, the analog signal output of the sensor was amplified 10 times and input into the data acquisition instrument. Thus, the sensitivity of the detection system to stress detection was about 0.7 mv/MPa. As resolution of the sensor is 0.1 mOe, its detection resolution to stress was 0.0353 MPa (0.1 mOe/2.83 mOe/MPa). In general, the above-mentioned detection sensitivity and resolution can satisfy internal stress detection requirements for strip steel during industrial production. Based on the analysis in this section, the designed excitation system and magneto-resistive sensor used in the detection device experimental process are shown in Table 4.

## 4. Analysis of Stress Detection Experiment and Influence Factor

Stress detection error and repeatability of the detection system were analyzed using the designed experimental device. Furthermore, the effects of strip thickness, carbon content, and crystal orientation were analyzed using this experimental device in this section.

### 4.1. Stress Detection Error and Repeatability

**Figure 7:**
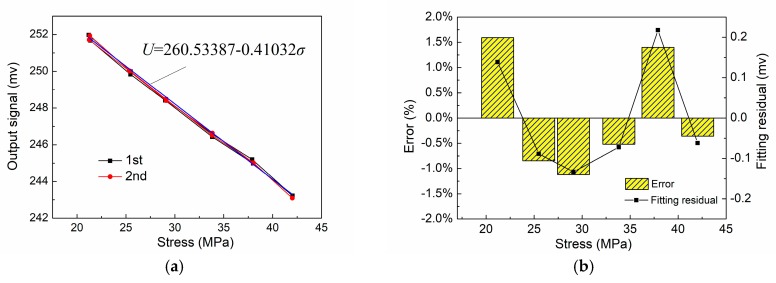
Detection error and repeatability analysis: (**a**) stress detection signal and fitting straight line; (**b**) detection error and fitting residual.

Figure 7a shows the output voltage signals at different internal stresses. The detected Q235 was two mm thick. The exerted stress range varied between 20 and 45 MPa. Two cyclic loading experiments were conducted for the same detected strip steel to evaluate repeatability. As shown in Figure 7a, the output signal of the detection system decreases with increasing internal stress for strip steel. In addition, excellent consistency was observed between output signals of two repeated experiments. According to the data statistics, the repeatability error of the detection system was 4% with a hysteresis error of 3.6%. Through the linear fitting of the least square method for output results, the internal stress of strip steel and the output signal of the detection system are in good linear relationship with a stress detection sensitivity *k* of 0.41032 mv/MPa. Figure 7b shows that the maximum fitting residual error of linear fitting is 0.21 mv with a linearity error of 2.5% for this detection system. The detection value of the system can be calculated based on the fitted equation. Detection error of the system (Figure 7b) can be obtained by using the detection value to subtract the actually measured standard stress value of the strain gage to divide by standard stress value at that time, which indicates that the maximum detection error of the system was about 1.5%. Therefore, the contactless stress detection principle can be used to conduct accurate internal stress detection for strip steel. Moreover, its detection precision was able to meet actual production requirements for strip steel.

### 4.2. Influencing Factors of Stress Detection Signals

For the practical production of strip steel, the main objective of detecting internal stress is to ensure the uniform distribution of internal stress at all positions to subsequently guarantee the final strip shape. Therefore, precisely measuring the internal stress differences at different positions on the same strip steel is the most important requirement for stress detection rather than obtaining the absolute value of internal stress. To accurately measure the difference in internal stress, the sensitivity of the detection signal must be accurately calibrated. Therefore, the effects of different influencing factors on signal sensitivity of stress detection are mainly discussed in this section.

**Figure 8:**
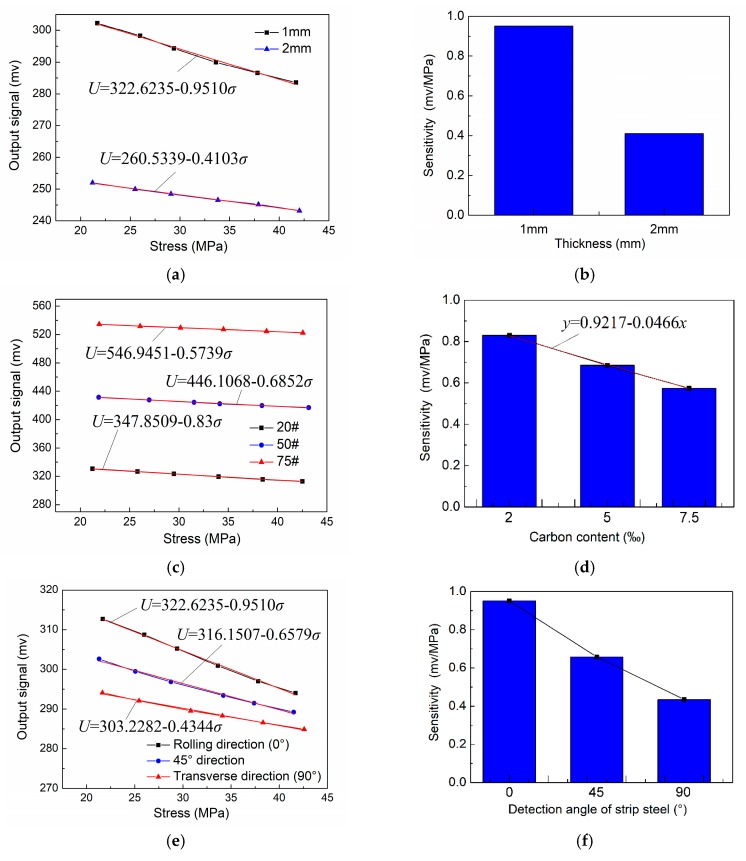
Analysis of influence factor for stress detection signal: (**a**) detection signal of strip steel with different thickness; (**b**) relationship between plate thickness and signal sensitivity; (**c**) detection signal of strip steel with different carbon contents; (**d**) relationship between carbon content and signal sensitivity; (**e**) detection signal in all directions of strip steel; and (**f**) relationship between different directions of strip steel and signal sensitivity.

**Figure 9:**
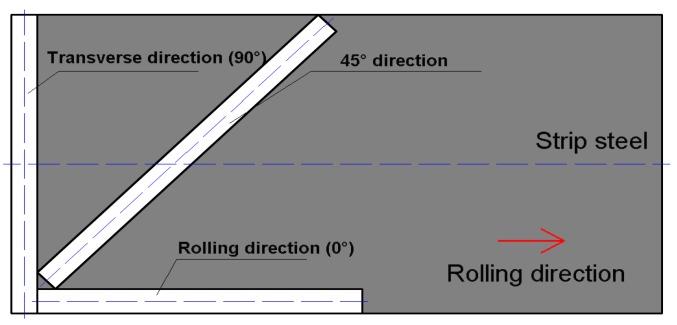
Analysis of influence factor for stress detection signal.

Figure 8a shows the detected results of strip steel with different thicknesses: one mm and two mm. The range of internal stress was 20 to 45 MPa. The stress detection was performed along the rolling direction under the same detection conditions listed in Table 4. As can be seen in this figure, the output value of the one mm detection signal was greater than that of the two mm strip steel. The sensitivity of the stress detection signal sharply decreased, which indicates that the internal stress of strip steel with a decreased thickness value is sensitive to the exciting field. In terms of strip steel with a greater thickness, the excitation parameters of the system, such as exciting current, should be increased to increase the sensitivity of the detection system. Strip steel with a thickness of one mm with different carbon contents, 2‰ (20# steel), 5‰ (50# steel), and 7.5‰ (75# steel), were subjected to a stress detection experiment along the rolling direction under the same detection conditions listed in Table 4. The results are shown in Figure 8c,d. The output value of the detection signal under the same stress level increased with an increase in strip steel carbon content. However, the sensitivity of the detection signal gradually decreased. Moreover, a monotonically decline relationship exists between sensitivity and carbon content. The sensitivity of stress detection decreased by 0.0466 mv/MPa with a 1‰ increase in strip steel carbon content. Therefore, to reach a certain stress detection sensitivity in the actual production process, the carbon content of a specific detected strip steel must be considered. In the production of strip steel, material undergoes considerable cold plastic deformation during cold rolling. The crystal form texture along the plastic deformation direction and preferred orientation of the crystal with certain directions were generated. The preferred orientation of the crystal significantly influences the magnetic anisotropy of ferromagnetic materials [30]. Although the internal stress detection of strip steel during industrial production is performed along the rolling direction, research on characterizing the stress detection signal for strip steel in different directions still has general theoretical importance and contributes to guiding the design of detection systems. Specimens of detected strip steel were cut on Q235 strip steel with the same thicknesses of one mm, and material along the rolling direction (RD), transverse direction (TD), and 45° (Figure 9) were subjected to stress detections under the same detection conditions listed in Table 4. The results are shown in Figure 8e,f, which show that the specimen of strip steel along the RD had the maximum output value for the detection signal and the maximum sensitivity. The transverse strip steel specimen had the lowest output value for detection signal and the least sensitivity; the output value and sensitivity the strip steel specimen along 45° was medium. In comparison with thickness and carbon content, although the output value of the detection signals for the three specimens had no considerable difference (the maximum output value was 315–290), the sensitivity of the same strip steel in different directions differed significantly. Therefore, stress detection direction must be strictly guaranteed along RD in actual detection processes. Otherwise, certain error may be generated due to the sensitivity of strip steel along different directions. We showed that the sensitivity is significantly dependent on the crystal orientation of the stress detection signal. The crystal orientation degree is related to the production process parameters of the strip steel, such as rolling reduction and annealing temperature [30,31,32]; therefore, the stress detection system must be recalibrated when the production process parameters of the strip steel with the same thickness and carbon content are changed. Finally, the experimental results showed that the detection principle can be used to detect internal stress and identify crystal orientation information of materials, so the detection principle could potentially be applied to detect crystal orientation through magnetic anisotropy.

## 5. Conclusions

This study proposed a novel contactless stress detection technology for cold-rolled strip steel based on a magneto-resistive sensor and the magneto-strictive effect that satisfy industrial requirements for accurate detection of strip steel internal stress. Our main conclusions were drawn as follows. 

Theoretical analysis indicated that the internal stress of strip steel and the voltage output of magneto-resistive sensors are linearly related. The sensitivity of stress detection is related to the magnetic characteristics of the detected strip steel, exciting parameters, and sensor parameters. The sensitivity of stress detection can be adjusted by changing some of the parameters. The main flux direction of the exciting field, the detection direction of the magneto-resistive sensor, and the length direction of the strip steel should be uniform so the maximum sensitivity for stress detection is obtained by the whole detection system.

An experimental device for stress detection was designed. The exciting parameters of the experimental device and sensor model were determined through numerical simulation. The stress detection experiment indicated that the proposed detection technology has good repeatability and linearity. Moreover, the detection error of the system was controlled at about 1.5%.

The intrinsic influencing factors of the detected strip steel on the sensitivity were explored. The sensitivity of the stress detection signal using a two-millimeter-thick strip steel was lower than that of a one-millimeter strip steel. The sensitivity of the stress detection signal decreased with increasing strip steel carbon content. The strip steel along RD had the highest stress detection sensitivity, whereas strip steel along TD had the lowest stress detection sensitivity. The stress detection system must be recalibrated when the production process parameters of strip steel are changed.

## Figures and Tables

**Table 1:** Signal conversion coefficient and influential parameters.

Signal Conversion Process	Conversion Coefficient	Influencing Parameter
Δ*σ→*Δ*μ*	$k_{\sigma -\mu }$	Saturation induction $B_{m}^{\prime }$ of strip steel materials; Saturated magneto-strictive coefficient *λ_m_* of strip steel materials; Magnetic permeability $\mu_{0}$ of strip steel materials under unstressed conditions
Δ*μ→*Δ*Bs*	$H_{s}$	Number of turns of excitation coil *N*; Excitation current *I*; Material and structure of excitation coil; Clearance *l_a_* between magnetic core and magnetic core
Δ*Bs→*Δ*Br*	${\left(a_{s-s^{\prime }}\right)}^{d}$	Vertical distance *d* between sensor detection point and strip steel plane
Δ*Br→*Δ*R*	$k_{B_{r}-R}$	Type and performance of magnetoresistance sensors
Δ*R→*Δ*U*	$U_{in}/R$	Supply voltage *U_in_* of sensor; Resistance value *R* of magnetoresistance sensors

**Table 2:** Mechanical parameters and magnetic characteristic parameters of simulated strip steel.

Parameter	Value	Parameter	Value
Young modulus E(GPa)	210	Electrical conductivity (S/m)	$4.032\times 10^{6}$
Poisson ratio	0.3	Electrical resistivity (Ωm)	$2.48\times 10^{-7}$
Density $\rho ({\mathrm{Kg}/m}^{3})$	7850	Saturation induction $B_{m}(T)$	2.5
Tensile strength $\sigma_{b}(\mathrm{MPa})$	370	saturation magnetostriction $\lambda_{m}$	$5\times 10^{-6}$
Yield strength $\sigma_{s}(\mathrm{MPa})$	235	Initial permeability $\mu_{0}$	500

**Table 3:** Performance parameter of the TMR2102 sensor.

Parameter	Value	Parameter	Value
Linear region (mT)	±3.0	Operating temperature (°C)	−40–125
Sensitivity (mV/V/mT)	49.0	Maximum applied magnetic field (Oe)	1500
Input voltage range (V)	1–7	Resolution (mOe)	0.1
Operating frequency (Hz)	DC, 1 M	Nonlinearity (%)	2.0

**Table 4:** Exciting system and sensor parameter in experimental process.

Exciting Magnetic Core	U22 Manganese Zinc Ferrite	Model of Sensor	TMR2102
Exciting current (mA)	49.0	Vertical distance between detection point and strip steel (mm)	2
Number of turns of excitation coil	DC, 1 M	Materials of excitation coil	Enameled copper wire with 0.02 mm diameter
